# Inhibition of the anti-apoptotic protein BCL2 in EML4-ALK cell models as a second proposed therapeutic target for non-small cell lung cancer

**DOI:** 10.1371/journal.pone.0308747

**Published:** 2025-01-21

**Authors:** Richard Junior Zapata Dongo, Diletta Fontana, Luca Mologni, Juan Enrique Faya Castillo, Stefany Fiorella Infante Varillas

**Affiliations:** 1 Department of Basic Sciences, Bioethics and Human Life, Faculty of Human Medicine, University of Piura, Miraflores, Lima, Perú; 2 Department of Medicine and Surgery, University of Milano-Bicocca, Monza, Italy; King Faisal Specialist Hospital and Research Center, SAUDI ARABIA

## Abstract

The anaplastic lymphoma kinase (ALK) oncoprotein plays a crucial role in non-small cell lung cancer (NSCLC) by activating signaling pathways involved in cell proliferation and survival through constitutive phosphorylation. While first-line crizotinib can regulate phosphorylation, mutations in the ALK gene can lead to resistance against ALK inhibitors (ALKi) such as ceritinib and alectinib. On the other hand, overexpression of BCL2, a protein involved in cell death regulation, has been observed in NSCLC and is considered a potential therapeutic target. In this study, we propose to inhibit BCL2 as a secondary therapeutic target in EML4-ALK cell models to overcome resistance caused by ALK mutations. Four Ba/F3 EML4-ALK cell models (WT, C1156Y, L1196M, and G1202R) generated by site-directed mutagenesis exhibited varying levels of BCL2 expression. Both the WT and G1202R models showed overexpression of BCL2, while C1156Y and L1196M models approached baseline levels. We treated these cells with ABT-199, a selective BCL2 inhibitor, and found that models with high BCL2 expression exhibited resistance, while those with lower expression showed sensitivity to BCL2 inhibition. In addition, our analysis using bioinformatics indicated that ABT-199 not only targets BCL2 but also binds to the active site of all ALK mutants, it was contrasted by *in vitro* ALK kinase activity inhibition by ABT-199 (5.5 μM). This interaction was further supported by a significant decrease of ALK phosphorylation in single and combination treatment with 300nM ABT-199. Finally, when ABT-199 was combined with ALKi, we observed a wide range of synergistic effects in the WT and G1202R cell models, while the C1156Y and L1196M models showed limited synergy. In conclusion, our findings indicate that BCL2 targeting with ABT-199, in combination with ALKi, can significantly reduce tumor cell survival in Ba/F3 EML4-ALK cell models.

## Introduction

Non-small cell lung cancer (NSCLC) accounts for 80% to 85% of all lung cancers (LC) diagnosed in the United States; it is estimated that about 224,580 adults (116,310 males and 118,270 females) will be diagnosed in 2024 [1]. Several oncoproteins involved in the development of LC have been identified, including KRAS (25%), EGFR (15–20%), ALK (2–8%), and others [2–4].

The Anaplastic lymphoma kinase (ALK) protein is a transmembrane protein encoded by the *ALK* gene located in the 2p23 region and has been identified in NSCLC carrying the paracentric inversion: Inv(2)(p21p23), involving *EML4-ALK* fusion gene [5]. In addition, ALK gene also has been involved in other kind of cancer such anaplastic cell lymphoma, breast cancer, melanoma, and others [6], even new studies included to thyroid cancers [7, 8].

EML4-ALK mutation generates the loss of the extracellular domain of the ALK protein, leading to constitutive phosphorylation of the intracellular tyrosine kinase domain trapped into the cytosol, and to the activation of oncogenic signaling pathways such as cell survival, proliferation and tumor growth [9]. Food and Drug Administration (FDA) has approved first-generation tyrosine kinase inhibitors (TKIs) targeted to ALK namely crizotinib and second-generation inhibitors such as ceritinib, alectinib and brigatinib. These ALK inhibitors compete with ATP to regulate ALK constitutive phosphorylation as well as downstream signaling pathways [10]. On the other hand, amino acid point mutations in the ALK protein including C1156Y, L1196M, G1202R, and others, usually generate resistance to TKIs [11]. In fact, this has motivated the search for new molecules aimed to ALK mutations, that include PF-06463922, TSR-011, ASP3026, X-396, CEP-37440, and XMU-MP-5 with motivating results. However, it is important to note that the process of efficacy and safety testing, along with obtaining FDA approval, is a time-consuming endeavor. Considering this, exploring new strategies such as combining therapies with inhibitors targeting multiple therapeutic targets or exploring drug repositioning may provide valuable insights into combating resistance in NSCLC. For instance, drug repositioning has already identified up to 16 preexisting drugs that target transcription factors associated with NSCLC-related genes [12–14], current studies propose mitoxantrone and abacavir as ALK-targeting for NSCLC [15].

BCL2 antiapoptotic proteins (BCL2, MCL1 and BCLxl) are currently considered as therapeutic targets in many types of cancer, due to their overexpression and the ability to apoptosis evasion [16, 17]. Hence, BCL2 inhibitors such as navitoclax, obatoclax and venetoclax are considered as therapeutic strategies for regulation of BCL2 family proteins. Venetoclax, also known as ABT-199, is a selective BCL2 protein inhibitor approved by the FDA for the treatment of chronic lymphocytic leukemia as monotherapy therapy, and in combination therapies for other hematologic malignancies [18, 19]. In addition, it seems likely that this strategy could play an important role in solid tumors [20].

Expression of BCL2 evaluated in patients with NSCLC inversely correlated with survival [21]. Likewise, other studies have shown that the overexpression of BCL2 in NSCLC may lead to resistance to treatment with anti-EGFR inhibitors (AZD9291) [22–24]. Based on this, we propose that BCL2 protein could be considered as a second therapeutic target in NSCLC, because the overexpression of this protein has been evidenced; ABT-199 could be considered as candidate inhibitor to improve this strategy as it was evidenced its synergistic capacity in combination with anti-EGFR in cellular models of NSCLC [23]. Furthermore, crizotinib, ceritinib, and alectinib are used in the standard treatment for NSCLC in ALK-mutation-positive patients, so both drugs (ABT-199 and TKIs) would be good candidates to work together. Consequently, the aim of our study was to demonstrate that the BCL2 protein could play a very important role as a second therapeutic target in EML4-ALK cell models by inhibiting with ABT-199 in combination with TKIs.

## Materials and methods

### Cell models

Four cell models of non-small cell lung cancer (Ba/F3 EML4-ALK^WT^, Ba/F3 EML4-ALK^C1156Y^, Ba/F3 EML4-ALK^L1196M^ and Ba/F3 EML4-ALK^G1202R^) were generated by Site-directed mutagenesis by Fontana D. et al, and kindly provided for this research [25]. These cell models were maintained in RPMI-1640® culture medium, supplemented with 10% inactivated fetal bovine serum, 1% penicillin/streptomycin, incubation at 37°C and 5% CO_2_.

### ALK and BLC2 Inhibitors

ALK inhibitors crizotinib, ceritinib, alectinib and BCL2 inhibitor venetoclax (ABT-199) were obtained commercially from Medchem express-MCE®. These drugs were resuspended in dimethyl-sulfoxide and stored at -20°C.

### Cell viability

10^5^ cells/mL of each cell model (Ba/F3 EML4-ALK ^WT^, Ba/F3 EML4-ALK ^C1156Y^, Ba/F3 EML4-ALK ^L1196M^ and Ba/F3 EML4-ALK ^G1202R)^ were cultured in 96-well plates per triplicated and exposed to the drugs (crizotinib, ceritinib, alectinib and ABT-199) in eight increasing concentrations up to 10 μM. After 48 hours of pharmacological treatment, the cell models were exposed with the 10% CellTiter-Blue® reagent, incubated for 3 hours and subsequently monitored in Multiskan Go spectrophotometer (ThermoScientific®) at a wavelength of 490nm. The data collected allowed us to calculate the Half-maximal inhibitory concentration (IC_50_).

### *In vitro* treatments

10^6^ cells/ml of each cell model were cultured per duplicated in different 12-well plates. In each dish a cell model was cultured and exposed to four different conditions (control: no treatment, 100 or 300 nM Crizotinib, 50 nM Ceritinib and 30 or 50 nM Alectinib and 300 nM ABT-199). After 24 and 48 hours of treatment, the cells were collected and lysed with laemmly buffer (1X) at 97°C for 20 minutes; subsequently stored at -20°C.

### BCL2 and ALK phosphorylation expression by western blot

Cell lysates maintained in laemmly buffer (1X) were loaded in SDS polyacrylamide gel wells and transferred to the nitrocellulose membrane. Subsequently, this membrane was blocked with fat-free milk (5%) for 1 hour, and immediately treated with the primary antibody: BCL-2 antibody: Bcl-2 (50E3) Rabbit mAb #2870 (Cell Signaling Technology), p-ALK: Phospho-ALK (Tyr1604) Antibody #3341 (Cell Signaling Technology), ALK: ALK (31F12) Mouse mAb #3791 (Cell Signaling Technology) and Actin: Anti-Actin antibody #A2066 (Sigma-Aldrich), both incubated overnight at 4°C. The next day the membrane was exposed to the secondary antibody Anti rabbit: Goat Anti-Rabbit IgG (H + L)-HRP Conjugate #1706515 (Bio-Rad®) or Anti mouse: Goat Anti-Mouse IgG (H + L)-HRP Conjugate #1706516 (Biorad) for 1 hour, finally, it was revealed and exposed in the Chemidoc Imaging Instrument (Bio-Rad®), where the expression bands were evidenced, and bands-quantification were analyzed by Image Lab 6.1 software (Bio-Rad®).

### Kinase assay

*In vitro* inhibition of ALK kinase by ABT-199 was evaluated using an ELISA-based kinase assay developed internally, using purified recombinant GST-ALK and a peptide substrate, as described previously by Mologni L, et al [26].

### Synergy assay

In a 96-well dish, 10^5^ cells/mL of each cell model were exposed per duplication to the drug combination of crizotinib, ceritinib or alectinib with ABT-199, based on independently calculated IC_50_ values. The pharmacological synergy was evidenced with the 10% CellTiter-Blue® reagent, after 3 hours it was monitored in Multiskan Go spectrophotometer (ThermoScientific®) at a wavelength of 490 nm.

### Apoptosis assay

Apoptosis detection was performed by flow cytometry using the eBioscience™ Annexin V Apoptosis Detection Kit (Thermo Fisher). Ba/F3 EML4-ALK^WT^, Ba/F3 EML4-ALK^C1156Y^ and Ba/F3 EML4-ALK^L1196M^ cells were seeded into a 6-well plate, and drugs were added at the indicated concentrations (No treatment, 300 nM ABT-199, 300 nM crizotinib, 300 nM ABT-199 + 300 nM crizotinib, 50 nM ceritinib, 50 nM ceritinib + 300nM ABT-199, 30 nM alectinib, and 300 nM ABT-199 + 30 nM alectinib). After 48h treatment, 5x10^5^ cells were harvested, washed once in phosphate buffered saline (PBS) at 4°C, and resuspended in 200 μL Binding buffer (1x). Annexin V-FITC and Propidium Iodide (PI) were added according to the manufacturer’s instructions. Flow cytometry analysis was conducted using an AttuneTM NxT Flow Cytometer (Thermo Fisher).

### Preparation of three-dimensional structures of ALK^wt^ and ALK^mutants^

ALK structures were prepared by molecular modeling and downloading of Alpha-fold template. Molecular modeling was performed in Yasara™ (Yet Another Scientific Artificial Reality Application) software, version 23.9.29 [27]. The amino acids sequence of the ALK protein (1620 aa) was obtained from the free repository UNIPROT (Code: Q9UM73), and only was modeled the catalytic domain (cd) of ALK by homology taking as reference five proteins stored in RCSB-PDB (Code: 5AAA, 4MKC, 3AOX, 6MX8 and 5AA9), this new ALK structure we named cdALK^WT^. Additionally, we also downloaded ALK structure from AlphaFold (AF) with the code AF-Q9UM73-F1 then we isolated the catalytic domain for next experiments, this new ALK structure we named AF-ALK^WT^, both ALK structures were saved in “.pdb” format. Subsequently, these three-dimensional structures ALK^WT^ (both cd and AF) were deliberately mutated at position 1156 Cysteine, 1196 Leucine, and 1202 Glycine of the amino acid sequence of ALK^WT^, for generate point amino acids mutations performing a change of a cysteine for tyrosine, leucine for methionine, and glycine for arginine respectively, under conditions of minimized energy (parameter assigned in the Yasara™ software). These new structures ALK^Mutant^ (cd and AF) also were saved in ".pdb" format.

### ALK^mutant^ receptor and ABT-199 ligand Molecular Docking

It was carried out within the parameters of the YASARA™ program. These new three-dimensional structures ALK^WT^, ALK^C1156Y,^ ALK^L1196M^ and ALK^G1202R^ (both cd and AF) were used as a substrate for molecular docking with ATP (natural ligand), crizotinib, ceritinib, alectinib and ABT-199 ligands. Molecular docking was performed between each ALK^WT^, ALK^C1156Y,^ ALK^L1196M^ and ALK^G1202R^ receptor (cd and AF) with the specific inhibitor ABT-199 (ligand isolated from 6O0K pdb), as well as crizotinib (isolated from 5AAA pdb), ceritinib (isolated from 4MKC pdb) and alectinib (isolated from 3AOX pdb). These new structures ALK^mutants^ (cd and AF) were saved in ".pdb" format and submitted to Protein-Ligand Interaction Profiler (PLIP) web to visualize the interactions between ABT-199 and amino acids of ALK active site classified in hydrophobic and hydrogen bond interactions groups [28].

### Binding energy calculation of protein-ligand interactions

The binding energy of protein-ligand interactions was measured independently in the YASARA™ program, where the manual indicates that the most positive energies show a better protein-ligand binding [29]. To do this, the protein-ligand under study was imported in ".pdb" format, followed by the removal of water molecules and separation of objects and molecules with the command ">splitObj X" (where "X" is the number assigned by the program for the object of interest). For binding energy calculation, we proceeded with the command ">BindEnergyObj Y" (where "Y" is the number assigned by the program for the ligand of interest to be measured). Finally, the energy calculated by the software was measured in kcal/mol.

### Statistical analysis

The statistical analyses were carried out in the software GraphPad Prism version 10.0.3 and Combenefit 2.02 for Windows 64x, these experiments were carried out in duplicate/triplicate, taking as analysis data the mean average ± the standard deviation (SD).

Cell survival were normalized in percentage (control cells = 100%) [30], the calculation of the IC_50_ was performed using parametric nonlinear regression adjusted to a 95% confidence interval [31]. Meanwhile, the comparison by multiple groups of variables was used Anova and turkey test. Finally, for the calculation of the combination index and synergy scores (Bliss, Loewe and HS) were calculated by free download Combenefit 2.02 software (https://www.cruk.cam.ac.uk/research-groups/jodrell-group/combenefit), to determine the effect of two drugs working together [32].

## Results

### Ba/F3 EML4-ALK^mutant^ cell models exhibit variable doses-response to ABT-199 and heterogeneous expression of BCL2 protein

**Fig 1:**
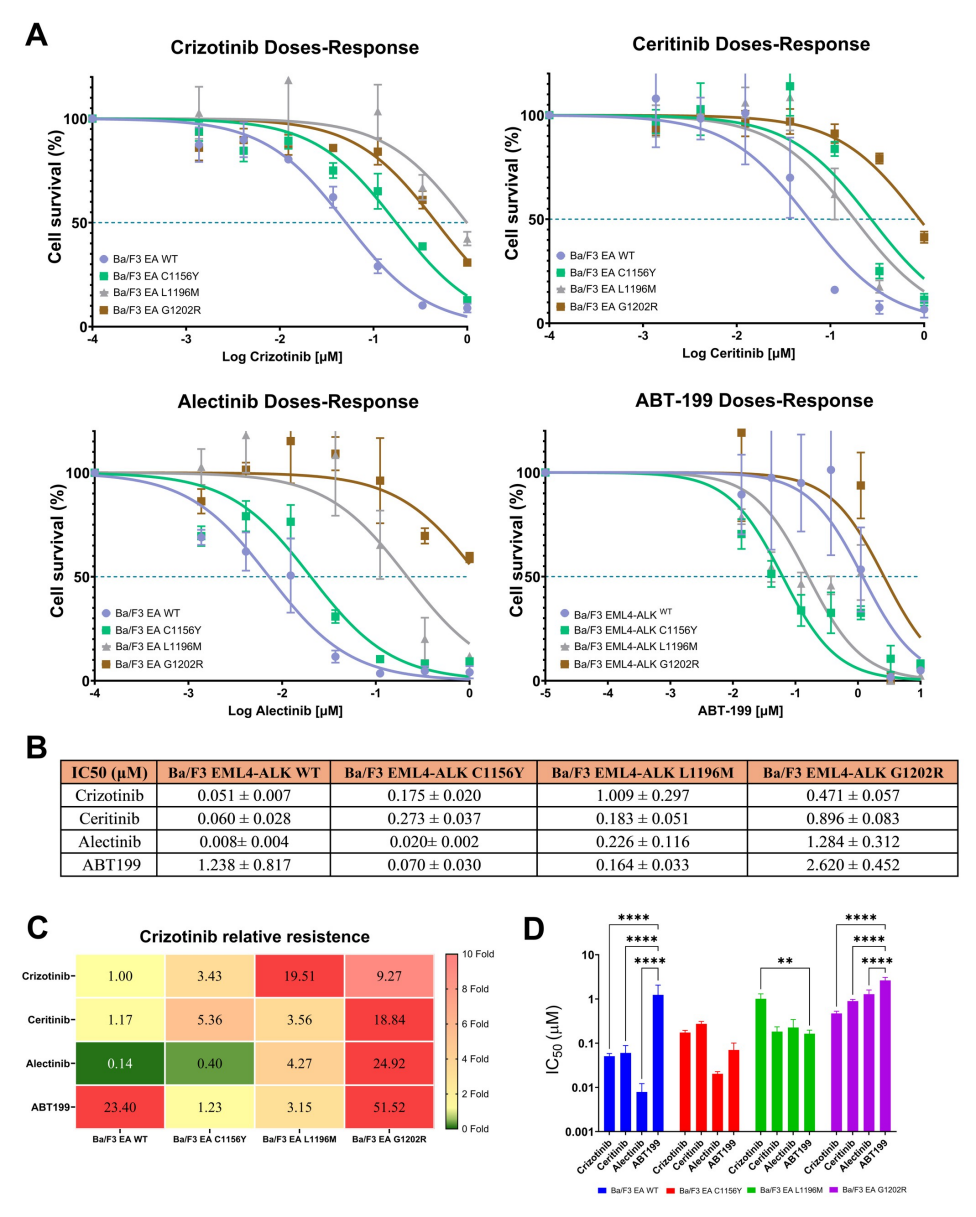
Ba/F3 EML4-ALK^mutants^ exhibit variable dose-response to ABT-199 treatment. Cell survival dose-response curves of Ba/F3 EML4-ALK^mutants^ (WT, C1156Y, L1196M and G1202R) treated with increasing concentrations of ALK inhibitors (crizotinib, ceritinib and alectinib) and BCL2 inhibitor ABT-199. B) Table shows IC_50_ values (Mean ± SD) of crizotinib, ceritinib, alectinib and ABT199 treatment in four Ba/F3 EML4-ALK^mutants^ cell models, calculated by parametric nonlinear regression in GraphPad Prism. C) The heat map shows the relative resistance to treatments taking crizotinib in WT cells as a reference; these values were calculated as the average IC_50_ ratio between all treatments in Ba/F3 EML4-ALK^mutants^ and crizotinib treatment in Ba/F3 EML4-ALK^WT^. The data show that EML4-ALK^WT^ and EML4-ALK^G1202R^ are resistant to ABT-199 treatment, while EML4-ALK^C1156Y^ and EML4-ALK^L1196M^ are more sensitive. D) Bars representation of IC50 values in the different cell lines treated with the various inhibitors. Statistical comparisons were done by Anova and Tukey test; asterisks indicate significance: p< 0.05 (*p<0.05; **p<0.01; ***p<0.001; ****p<0.0001).

Ba/F3 EML4-ALK^mutant^ cell models were evaluated by dose-response treatment with increasing concentrations of ALK inhibitors (TKIs): crizotinib, ceritinib and alectinib; and ABT-199, to calculate the IC_50_ values. Our results highlight that WT (IC_50_: 1.24 ± 0.82 μM) and G1202R (IC_50_: 2.62 ± 0.45 μM) were strongly resistant to ABT-199 treatment (p<0.0001), while C1156Y (IC_50_: 0.070 ± 0.03 μM) and L1196M (IC_50_: 0.164 ± 0.03 μM) appear to be sensitive to ABT-199; in the L1196M model ABT-199 proved to be more active than crizotinib, due to fact that this mutant is known to be resistant to crizotinib (IC_50_: 1.00 ± 0.29 μM) (Fig 1).

**Fig 2:**
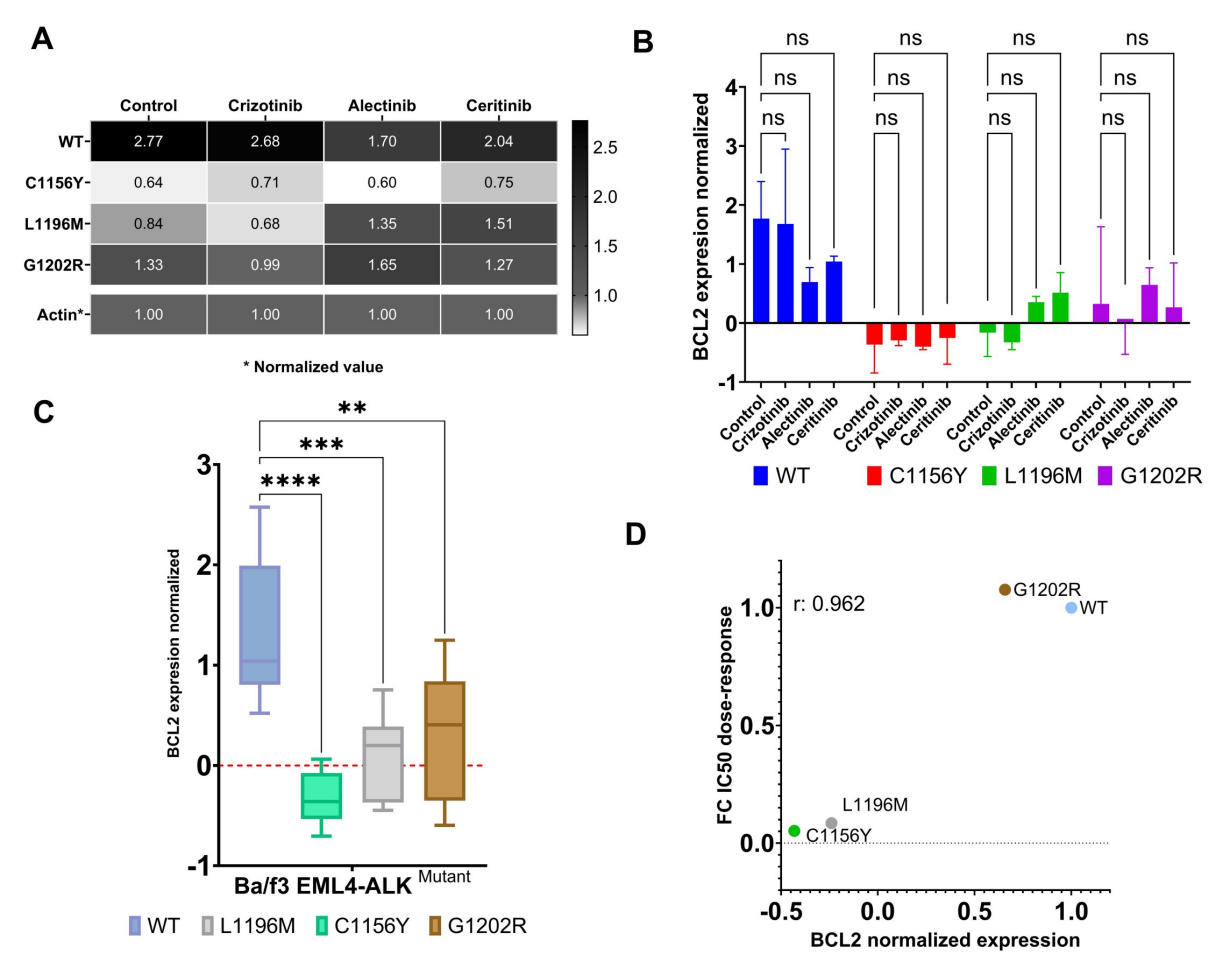
BCL2 antiapoptotic protein exhibits heterogeneous expression in Ba/F3 EML4-ALK^mutants^. A) Heatmap show the expression of BCL2 in different treatment conditions: control, 100nM crizotinib, 50nM ceritinib, and 50nM alectinib, and at two different time points (24 and 48 hours). No differences in BCL2 expression are evident between the two time points. B) Graph bars show BCL2 expression normalized using β-actin as loading control at 48 hours. ANOVA with Tukey test is used for statistical comparisons C) Box plots show in summary the average of BCL2 expression in ALK mutants across all treatment conditions; WT cell model overexpressed BCL2 followed by G12022R, while C1156Y and L1196M exhibited BCL2 expression closely to baseline. D) Pearson correlation analysis between IC_50_ values and BCL2 expression levels (r: 0.962 and p: 0.0332). Overexpression of BCL2 exhibits resistance to ABT-199, while baseline expression shows sensitivity to ABT-199. Statistical values: significant p< 0.05 (*p<0.05; **p<0.01; ***p<0.001; ****p<0.0001).

BCL2 expression measured in patients and NSCLC cell lines evidenced high expression that it could be responsible for resistance to novel molecules of treatments [21, 22]. Then, we hypothesized that BCL2 could be overexpressed in EML4-ALK cell models as well. Based on this, BCL2 protein expression was measured after 24 and 48 hours in four EML4-ALK cell models (WT, C1156Y, L1196M and G1202R mutations) in different treatment conditions: Control (no treatment), crizotinib (100nM), ceritinib (50nM) and alectinib (50nM); these concentrations were obtained from IC_50_ dose-response calculation. Our results showed heterogeneous BCL2 expression: control condition evidenced high expression of BCL2 in WT model, followed by G1202R, while C1156Y and L1196M showed low BCL2 expression. The same outcome was evidenced in 100nM crizotinib condition, and finally, 50nM ceritinib and 50nM alectinib condition does not evidence significative differences (Fig 2A and 2B. In addition, we can see that four cell models despite exhibit variable BCL2 expression, these keep their expression in the different condition treatment (S1 Fig). In general, regardless of the treatment, WT and G1202R mutations evidenced high expression of BCL2, while C1156Y and L1196M evidenced lower expression (Fig 2C). These BCL2 expression findings correlated with ABT-199 dose-response data, and might explain different sensitivity to the drug (Fig 1A). This correlation evidenced that WT and G1202R need high doses of ABT-199 to inhibit BCL2 overexpression, while C1156Y and L1196M exhibited sensitivity to ABT-199 due to low expression of BCL2 (Fig 2D).

In summary, our results suggest that there is a codependence and correlation between BCL2 expression and the pharmacological susceptibility to ABT-199 (Pearson coefficient = 0.962; p = 0.0378). In some cases, such as C1156Y and L1196M mutant, ABT-199 showed the same efficacy as ALKi; it remains to be clarified whether ABT-199 could interact with ALK.

### High binding energies suggest that ABT-199 may fit into the active site of ALK mutations

Our viability data may be simply explained by ABT-199 targeting BCL2 protein in Ba/F3 EML4-ALK^mutant^ cells, or by an additional unpredicted effect on ALK activity. To verify this hypothesis, we docked ABT-199 to the ALK active site.

We know that ALK inhibitors including crizotinib, ceritinib and alectinib can fit into the catalytic domain of ALK because they compete with ATP molecule. That has been demonstrated in *in vitro* assays, clinical trials and in addition explained in *in silico* assays by the binding energy determination, where all ALK inhibitors exhibit notably larger binding energy than ATP [15]. Hence, we modeled the catalytic domain of ALK (cdALK) as described in methods, because we found some amino acids missing in the tridimensional structure crystalized in the RSCB-PDB repository. In addition, we downloaded ALK structure from alpha-fold (AF-ALK). Subsequently, these ALK structures were mutated in C1156Y, L1196M and G1202R amino acids, and all ALK mutants were docked with ABT-199; afterward we proceeded to the binding energy calculation, using PLIP to visualize the amino acids interactions.

**Fig 3:**
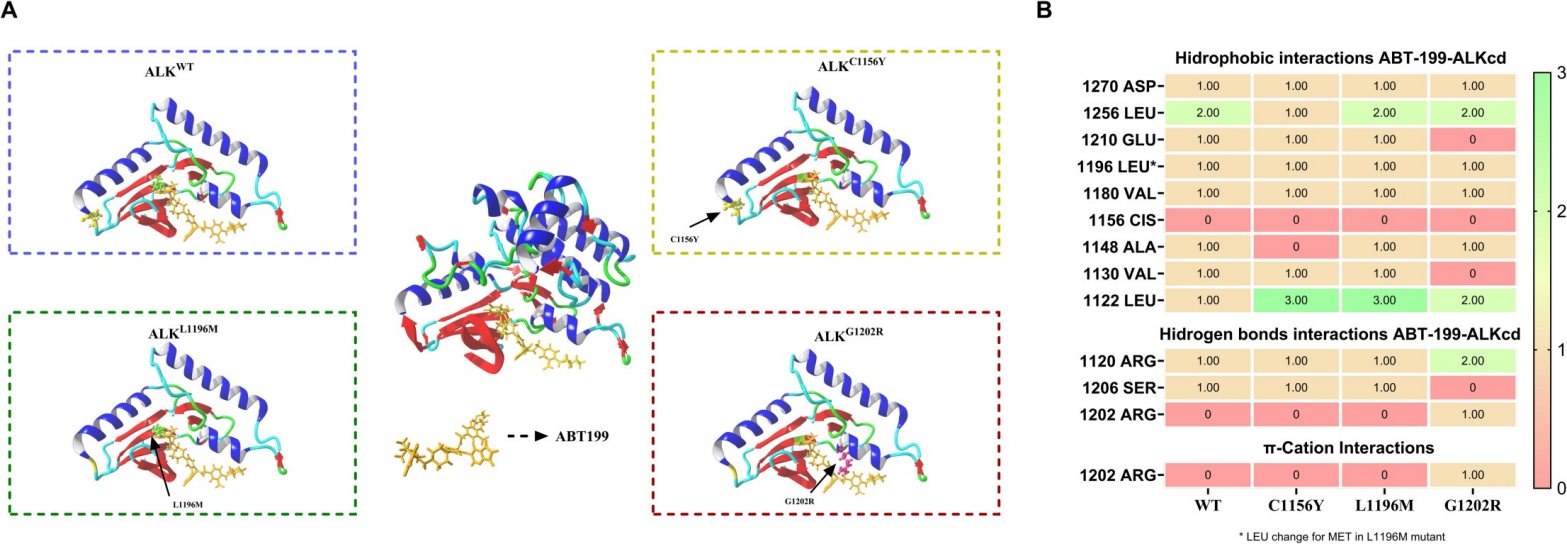
Molecular docking showed that ABT-199 can fit into active site of ALK^Mutant^. A) At the center of figure, the tridimensional structure of molecular docking of ALK^Mutant^ receptor interacting with ABT-199 ligand (orange) simulated in Yasara software is shown. Blue square zoomed ALK^wt^ binding to ABT-199: in yellow sticks we can see Cys1156, in green Leu1196 and in red Gly1202 amino acids (please see S1 for further details); Yellow square zoomed ALK^C1156Y^ binding to ABT-199: in yellow sticks we can see Tyr1156 amino acid point mutation (please see S2 for further details); Green square zoomed ALK^L1196M^ binding to ABT-199: in green sticks we can see Met1196 amino acid point mutation (please see S3 for further details); Red square zoomed ALK^G1202R^ binding to ABT-199: in red sticks we can see Arg1196 amino acid point mutation (please see S4 for further details); ABT-199 can fit into active site of ALK^Mutant^ by hydrophobic and hydrogen bond interactions. B) Heat map shows the number of interactions between cdALK amino acid positions and ABT-199 in all ALK^mutant^, classified into hydrophobic, hydrogen bonds, and π cation interactions. These data were obtained from PLIP website by prior submission “pdb.” of the molecular docking structure.

In this study, the ATP molecule was considered as a natural ligand for binding to cdALK and AF-ALK for phosphorylation simulation (Basal control), while ALK inhibitors (crizotinib, ceritinib and alectinib as a positive controls) were viewed as competing molecules for phosphorylation. Prednisone, used as negative control, docks outside of the ALK tyrosine kinase active site, with a strongly negative binding energy (-415.12 Kcal/mol). Additionally, ABT-199 was docked into ALK in order to investigate its potential interactions (Figs 3A and S2 and S3).

Structurally, we can see that ABT-199 is a larger molecule compared to ALK inhibitors, this does not prevent interaction with the ALK active-site in all ALK mutants (S2 Fig). This molecule can interact hydrophobically with important amino acids such as 1270 Asparagine; 1256, 1196 and 1122 Leucine; 1210 Glutamine; 1180 and 1130 Valine; and 1148 Alanine into the active-side of ALK. Additional interactions are established by hydrogen bonds (S4–S7 Figs) such as 1120 Arginine present in all ALK mutants, 1206 Serine (but not in G1202R mutant which however makes H-bonding with 1202 Arginine; this last interaction also exhibited π-Cation interactions). In these results we can appreciate something particular in the amino acid 1196, because it exhibits hydrophobic interaction with leucine, despite in L1196M mutant the amino acid change for methionine, this amino acid kept the hydrophobic interaction with ABT-199 in cdALK (Fig 3B).

**Fig 4:**
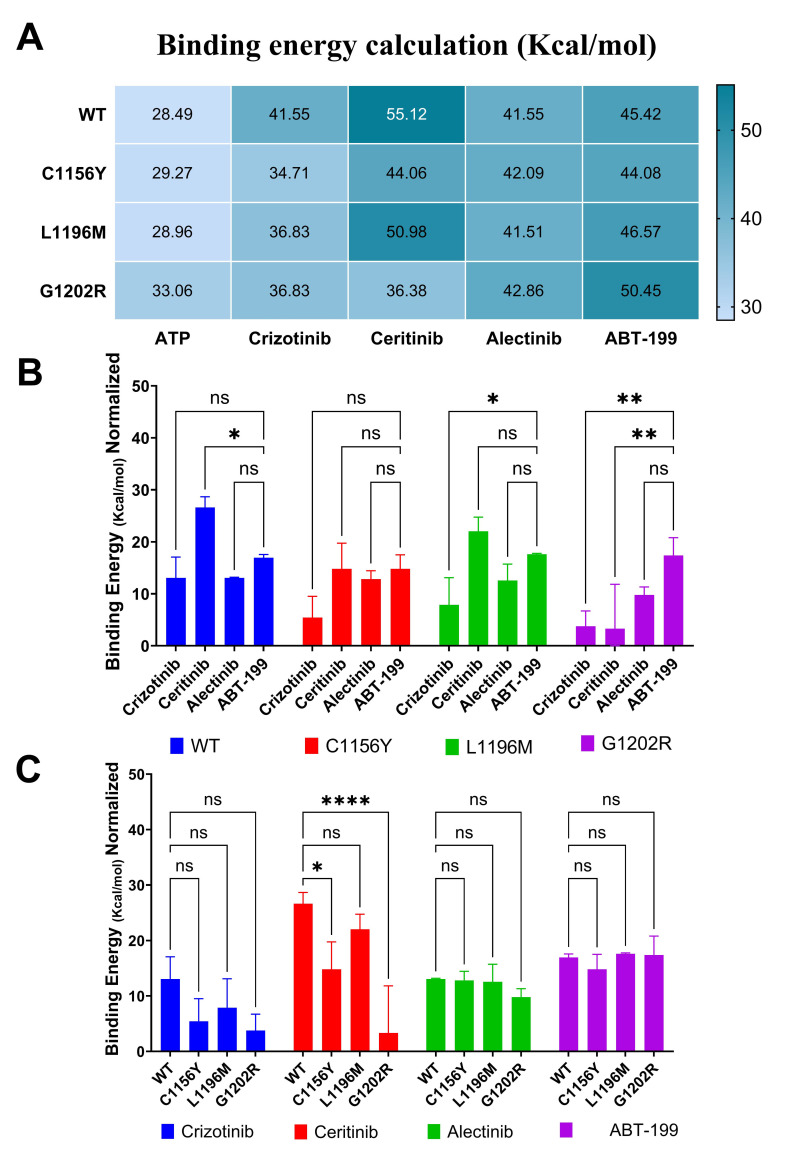
Amino acids interactions and binding energy describe that ABT-199 binds to ALK^Mutant^. A) Heat map shows the binding energy measured in Kcal/mol and calculated using YASARA™ software of ALK^mutant^ proteins (cdALK and AF-ALK) docked with ATP, crizotinib, ceritinib, alectinib, or ABT-199. Values represent the average of independent calculations using two different ALK models obtained from Yasara. B) Anova graph show binding energies mean values normalized with ATP, (grouped by mutant) here we can see to ABT199 competition with ALKi in each cell mutant model. C) Anova graph show binding normalized with ATP (grouped by ligand), here we can see that mutants exerts resistance to each ALKi, same case we can see that ABT199 do not exhibit significant differences in each ALK mutant. Statistical values: significant p< 0.05 (*p<0.05; **p<0.01; ***p<0.001; ****p<0.0001).

Binding energies calculated in Yasara™, in details we observed that G1202R (50.45 ± 0.43 Kcal/mol) and WT (45.42 ± 1.86 Kcal/mol) models showed high binding energy interacting with ABT-199 compared to ATP (33.06 ± 2.13 and 28.49 ± 1.54 Kcal/mol respectively), however, we can see in previous *in vitro* assays that they exhibit resistance to it. So, we hypothesize that ABT-199 at the used concentrations could not overcome elevated BCL2 expression in WT and G1202R. Other results show that ABT-199 could bind to ALK C1156Y active-site (44.08 ± 0.99 Kcal/mol), as all ALKi (crizotinib: 34.71 ± 0.30 Kcal/mol, ceritinib: 44.06 ± 0.14 Kcal/mol and alectinib: 42.09 ± 3.15 Kcal/mol), and finally, in L1196M mutation, ABT-199 can bind into ALK active-side (46.57 ± 2.25 Kcal/mol) and could compete with crizotinib (36.83 ± 0.29 Kcal/mol) and alectinib (41.55 ± 0.74 Kcal/mol) (Figs 4A and S8).

Independently, binding energies suggest that ABT199 cannot compete with ceritinib in WT (p = 0.0332) because it showed lower binding energy, while in C1156Y and L1196M does not exhibit significant differences (p = 0.1234) suggesting similar activity as ceritinib. Similarly, ABT199 showed no significant differences with crizotinib in WT and C1156Y, but was better in L1196M and G1202R (p = 0.0332 and p = 0.0021, respectively), while in comparison with alectinib not evidence significant differences in all mutants, and finally, as we demonstrated previously, this G1202R mutant is resistant to all ALKi and ABT199, then despite of it, our results showed that ABT199 could have more possibility to interacting with ALK because exhibited higher binding energy reference to crizotinib and ceritinib (p = 0.0021) (Fig 4B). Same time, these results allow us to corroborate that ALK mutants exhibited resistance to ALKi treatment and suggest that ABT199 can act with the same interaction than ALKi (Fig 4C).

In general, analyzing the binding energy consensus (the average energy from all ALK forms) we can see that ALKi compete directly with ATP for cdALK, as well as ABT-199 (p<0.0001). In addition, our findings suggest that ABT-199 could interact a little better than crizotinib and alectinib, nevertheless, it could not compete with ceritinib because the latter exhibits superior binding energy in all ALK mutants, except G1202R (p = 0.0001).

In summary, these findings showed that ABT-199 may fit into all ALK mutations active-site.

### Catalytic domain of ALK protein exhibited decreased phosphorylation due to interaction with ABT-199

**Fig 5:**
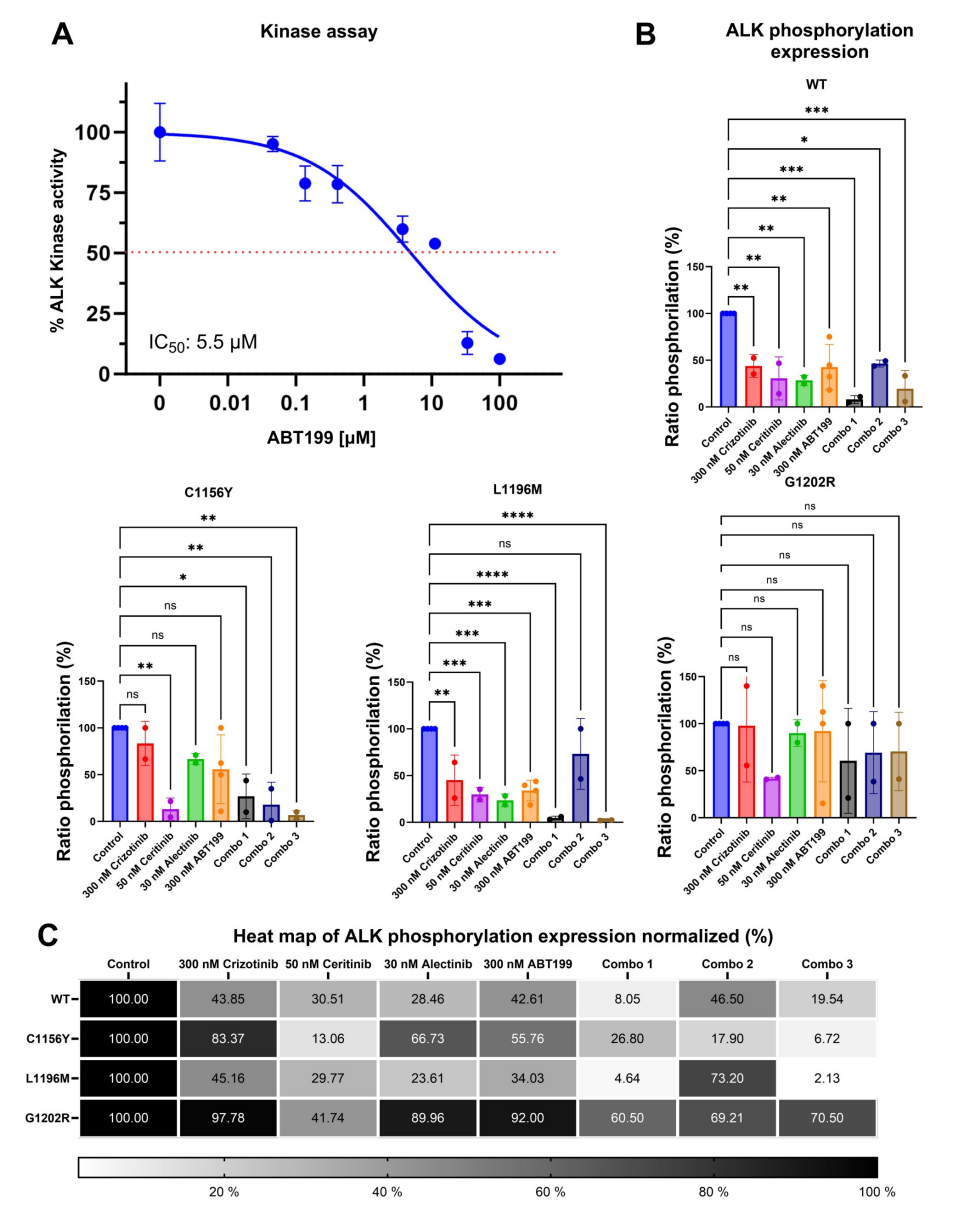
ABT199 inhibits ALK ^mutant^ kinase activity and decreases it phosphorylation. A) ALK kinase activity curve shows that ABT199 is able to inhibit ALK kinase activity (IC_50_: 5.5 μM). B) Graph bars show Anova and tukey test of pALK expression normalized to ALK. C) Heatmap show pALK expressions normalized after 48 hours treatment by ALKi or ABT199, and their combination (Combo 1: 300 nM ABT + 300 nM Crizotinib, Combo 2: 300 nM ABT + 50 nM Ceritinib, and combo 3: 300 nM ABT + 30 nM Alectinib). Statistical values: significant p< 0.05 (*p<0.05; **p<0.01; ***p<0.001; ****p<0.0001).

Our bioinformatics findings suggest that ABT-199 may bind to ALK. Then, we performed an *in vitro* kinase assay with purified recombinant ALK protein to verify direct enzymatic inhibition of ALK by ABT-199. The drug dose-dependently inhibited ALK kinase activity with an IC_50_ of 5.5 μM, thus confirming that the compound is able to block ALK by direct interaction. This finding suggests that ABT-199 may fit into ALK active site (Fig 5A).

To further confirm inhibition of pALK, we measured EML4-ALK phosphorylation in cells by western blot. Here, Ba/F3 EML4-ALK^mutants^ cell models were exposed to different treatment conditions: without treatment, crizotinib (300nM), ceritinib (50nM), alectinib (30nM) and ABT-199 (300nM). Phosphorylation was normalized over total ALK expression and untreated control was set to 100%. Our results in WT evidenced decreasing to 42.61% pALK (p = 0.0032) when it was treated with 300nM of ABT-199; in C1156Y mutant showed decreasing to 55.76% pALK (p = 0.0528); in L1196M exhibited decreasing to 34.03% pALK (p<0.0001); and finally, G1202R mutant did not show any significant decrease (p = 0.7763) of pALK (92%) (Figs 5B and 5C and S8).

Other results showed that phosphorylation in WT cells exhibited decreasing to 54% (300nM crizotinib), 30.51% (50nM ceritinib) and 28.46% (30nM alectinib) because this mutant is sensitive to all ALKi; C1156Y showed 83.37% (300nM crizotinib ‐ resistance), 13.06% (30nM ceritinib-sensitive) and 66.73% (30nM alectinib-sensitive); while L1196M evidenced 45.16% (300nM crizotinib-resistance), 29.77% (50nM ceritinib-resistance) and 23.61% (30nM alectinib-sensitive); and finally G1202R reveled 103.8% (300nM crizotinib), 41.74% (50nM ceritinib) and 89.96% (30nM alectinib) because this mutant is resistant to all ALKi (Figs 5B and 5C and S8).

On the other hand, pALK also was measured in combined conditions: combo 1 (300nM Crizotinib + 300nM ABT-199), combo 2 (50nM Ceritinib + 300nM ABT-199) and combo 3 (30nM Alectinib + 300nM ABT-199) in all four ALK mutant models. Our results showed strong decrease of phosphorylation in WT (residual phosphorylation combo 1: 8.05%; combo 2: 46.50%; and combo 3: 19.54%), C1156Y mutant (combo 1: 26.80%; combo 2: 17.90%; and combo 3: 6.72%) and L1196M mutant (combo 1: 4.64%; combo 2: 72.20%; and combo 3: 2.13%); finally, no or little effect was seen in G1202R mutant (combo 1: 63.09%; combo 2: 69.21%; and combo 3: 105.70%) (Figs 5B and 5C and S8). In summary, WT and C1156Y evidenced decrease of pALK in all combination treatments (p<0.05), in L1196M exhibited strong effectivity in the combo 1 and 3 (p = 0.0001), but not significant differences in combo 2, at last G1202R mutant showed no differences of phosphorylation in all combination treatments.

These results suggest that combination treatments could be used to achieve a synergistic response by dual therapeutic targeting.

### ALK inhibitors in combination with ABT-199 exhibited synergy dose-response in Ba/F3 EML4-ALK cell models

Previous results have identified ALK as a novel possible target of ABT-199. Then we proceeded to combine the BCL2 inhibitor ABT-199 with TKIs crizotinib, ceritinib and alectinib respectively in each Ba/F3 EML4-ALK cell models.

**Fig 6:**
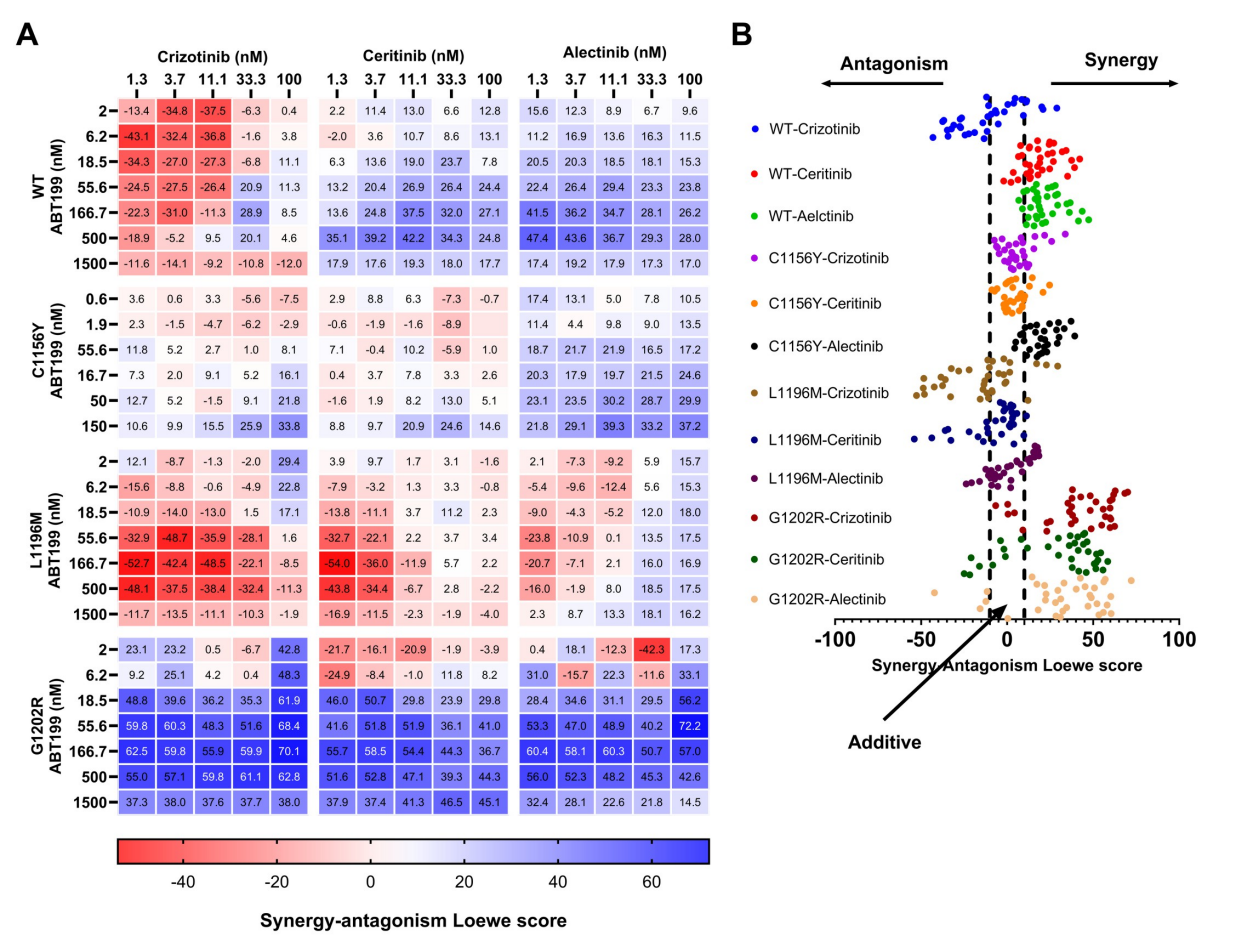
Loewe synergy-antagonism score analysis shows additive and synergistic effects after ALKi and ABT-199 combination treatment. A) Heat map shows the Loewe synergy-antagonism scores (synergy >10 “blue”, antagonism < -10 “red”, additive from -10 up to 10 “white”) in four EML4-ALK mutant cell models treated with ABT-199 in combination with ALKi (crizotinib, ceritinib and alectinib). B) Dot plot showed that WT and G1202R exhibit wide range of additivity and synergism, respectively, while C1156Y and L1196M showed limited range of synergism and wide range of additivity, respectively.

As we know, G1202R and WT models were resistant to ABT-199 (IC_50_: 1.238 ± 0.817 μM and 2.620 ± 0.452 μM respectively); however, surprisingly, ABT-199 in combination with crizotinib, ceritinib and alectinib showed synergistic inhibition of cell survival. The synergy Loewe scores (score synergy >10 “blue”, score antagonism < -10 “red”, score additive from -10 up to 10 “white”) evidenced to both WT and G1202R a wide range of synergism in all combinations. In this case we can demonstrate that despite of cell models mentioned above show resistance to ABT-199, in combination with ALKi we can decrease the cancer cell survival. On the contrary, C1156Y and L1196M showed sensitivity to ABT-199 (0.070 ± 0.030 μM and 0.164 ± 0.033 μM, respectively), but exhibited a wide range of additivity, and limited range of synergism, respectively (Fig 6A and 6B).

**Fig 7:**
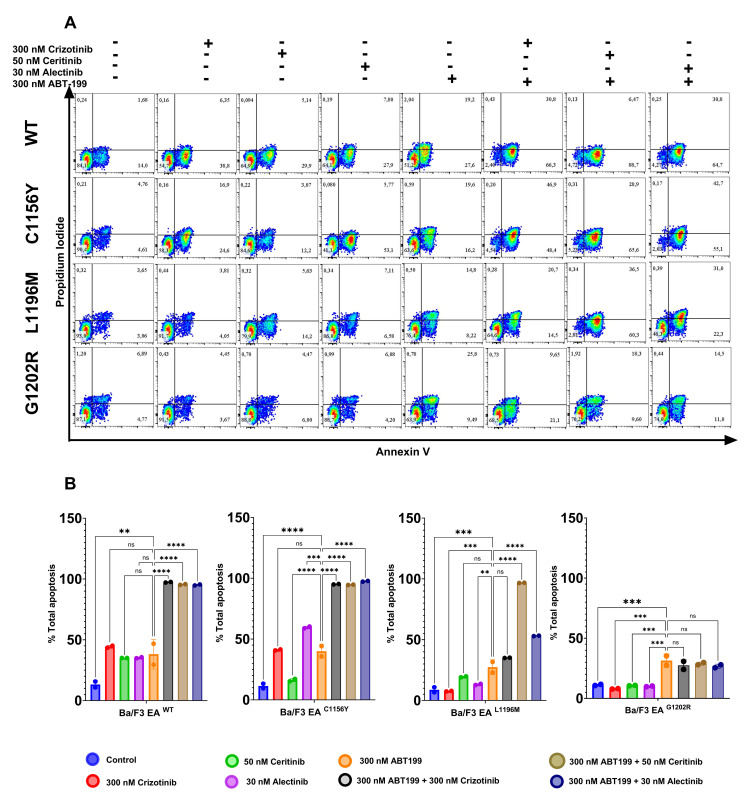
Combination therapy exhibited apoptosis in Ba/F3 EML4-ALKmutant cell models. A) Plots show apoptosis assay by flow cytometry, and B) graph bars show Anova and tukey test. Regarding WT, C1156Y, and L1196M mutants, these results show that combination treatments lead to apoptosis by synergistic and additive effects, as proved by Loewe analysis. In G1202R cells, there are no significant differences between ABT-199 and combination treatments. Statistical values: significant p< 0.05 (*p<0.05; **p<0.01; ***p<0.001; ****p<0.0001).

Afterwards, we proceed to measure the apoptosis assay in all four Ba/F3 EML4-ALK cell models in those three combination treatments (Combo 1; 2 and 3). In WT we evidenced 97.4% (p<0.0001) of apoptosis in combo 1; 95.10% (p<0.0001) (combo 2) and 95.49%(p<0.0001) (combo 3), in C1156Y: 95.65% (p<0.0001) (combo 1); 97.50% (p<0.0001) (combo 2) and 94.65% (p<0.0001) (combo 3), in L1196M: 35.05% (not significant; ns) (combo 1); 53.10% (p<0.0001) (combo 2) and 96.70% (p<0.0001) (combo 3); and finally G1202R: 27.43% (ns) (combo 1); 26.72% (ns) (combo 2) and 28.85% (ns) (combo 3). In summary, these results suggest strong conduction of apoptosis (p<0.0001) in all combination treatments of WT and C1156Y cell models, while L1196M cell model exhibited strong conduction of apoptosis in combo 2 and 3, however in combo 1 not evidenced significant differences respect to ABT-199 treatment (ns); same way in G1202R cell model we can observed conduction of apoptosis but with no significant differences respect to ABT-199 (ns) (Fig 7). Apparently G1202R model exhibited high synergy in proliferation assay while apoptosis assay can not demonstrate the same outcome. Indeed, the synergistic effect is more evident as an antiproliferative effect rather than pro-apoptotic one.

In summary, our results suggest that the combination of ABT-199 with ALKi could exhibit notable synergy in WT and G1202R models, C1156Y and L1196M exhibit a good synergy in combination with crizotinib and alectinib and additive with ceritinib.

## Discussion

NSCLC with chromosomal rearrangements in the ALK gene exhibit sensitivity to targeted therapies such as crizotinib, ceritinib, and alectinib. However, point amino acid mutations have been identified as responsible for exerting resistance to these drugs, including the L1196M mutation that causes resistance to crizotinib and in some cases to ceritinib and alectinib [33]; C1156Y is reported as resistant to both crizotinib and ceritinib; and finally G1202R showing resistance to all of three inhibitors: crizotinib, ceritinib and alectinib, and other mutations [34–36]. Based on this, cell models were chosen which were edited by site-directed mutagenesis with the following mutations C1156Y, L1196M and G1202R [25], these were exposed to dose-response with crizotinib, ceritinib and alectinib. The results obtained were as expected according to the literature, which assured us that these models would ideally represent NSCLC with mutations in the ALK gene.

On the other hand, the BCL2 protein has been identified three decades ago abnormally overexpressed in some lung carcinomas (37%), whose prognostic value would have an important impact [37]. Since 2000 to present, Hanahan and Weinberg have described the family of BCL2 proteins as hallmark of cancer and as a therapeutic target with mimicking molecules such as ABT-199, approved in 2015 for hematological malignancies as monotherapy and combination therapy [16]. In this study, we focused to BCL2 protein as a second therapeutic target for NSCLC since we found the overexpression of the BCL2 protein in WT and G1202R, while C1156Y and L1196M showed BCL2 closely to basal expression. When EML4-ALK cell models were exposed to ABT-199 treatment we can see that WT and G1202R exhibit resistance while C1156Y and L1196M evidenced sensitivity; these results were in accordance with the BCL2 expression in each cell line according to correlation analysis (Fig 2D). In vitro studies in LC showed that resistance to ABT-199 is due to the fact that other proteins belonging to the BCL2 family could also be overexpressed, including MCL1, which suggest as a dual therapeutic target to elucidate resistance [38]. Based on these studies, we would not rule out overexpression of MCL1 and BCL-xl in WT and G1202R models. Further in vivo studies revealed that ABT-199 showed sensitivity in LC with high BCL2 expressions [39]; from this study we infer that the L1196M and C1156Y models might just be overexpressing BCL2 protein in concentrations closely to basal expression and easily downregulated by ABT199, which would explain the sensitivity to ABT-199. These results would recommend investigating the other antiapoptotic proteins in NSCLC with ALK mutations.

Our computational study revealed that the ALK^L1196M^ model, modeled in Yasara, would have a better interaction energy with crizotinib (36.83 kcal/mol), compared to the first model ALK^L1196M^–crizotinib stored in RCSB database pdb code 2xp2 (30.86 kcal/mol), value calculated in Yasara™ [40]. Similarly, the interaction with alectinib (41.51 kcal/mol) also showed better value than the first model. ALK^L1196M^-alectinib PDB code 3AOX (27.77 kcal/mol) value also calculated in Yasara™ [41]. Our research team hypothesizes that this improved interaction energy is because the predictive modeling of the ALK protein was more complete because five reference ALK pdb models were taken (Code: 5AAA, 4MKC, 3AOX, 6MX8 and 5AA9), from which the missing regions of the protein’s three-dimensional structure could be completed. We suggest that ABT-199 could fit into ALK active-site, because it shares the same hydrophobic and hydrogen bond interactions with some important amino acids as ALKi (crizotinib, ceritinib, alectinib, brigatinib and lorlatinib) such as 1122Leu, 1130Val, 1148Ala,1180Val,1196Leu,1202Arg and 1256Leu that appears to be responsible to give stability to the ligand to the active site of ALK by hydrophobic interactions as reported by Faya Castillo J. et al [15]. In this study our results would indicate that all ALK models would be optimal for coupling with ABT-199 because there has been no literature on the interaction of this with ALK, whose calculated interaction consensus binding energy (average of all ALK forms) was 46.63 ± 2.77 kcal/mol being significative superior to ABT (p<0.0001; binding energy: 29.95 ± 4.17 kcal/mol) which would show that ABT-199 could inhibit the ALK protein, suggesting a dual targeting. BCL2 protein obtained from alphafold (AF-P10415-F1) in interaction with ABT-199 showed a better interaction energy (82.907 kcal/mol) than the pdb model: 6O0K (-50.6 kcal/mol) calculated in AutoDock Vina (its equivalent measured in Yasara™: 71.76 kcal/mol) [42]. In *In silico* and *in vitro* it has been shown that the ALK model can be inhibited by crizotinib, ceritinin, alectinib and ABT-199, suggesting ALKi and ABT-199 as combination therapy.

In combination therapy of TKIs with ABT-199 we have demonstrated that combo 1 (300nM crizotinib + 300nM ABT-199) was the better combination for decreasing the phosphorylation activity in all four Ba/F3 EML4-ALK cell models. For instance, we know that ALK WT was resistant to ABT-199 due to high expression of BCL2 protein then we need high concentrations to inhibit tumoral cell, however, ALK WT is sensitive to single crizotinib treatment, this is because crizotinib is a multitarget inhibitor such ALK, c-MET and ROS1 pathway [43], due to this crizotinib can lead to apoptosis to tumoral cell by inhibition of signaling pathways mentioned above, while single ABT-199 treatment is not enough to conduct tumoral cell to apoptosis due to BCL2 overexpression. Then we demonstrate that adding ABT-199 as second treatment could potentiate the conduction to apoptosis than single crizotinib treatment.

Currently, combination therapy is playing a leading role in drug resistance, for example ABT-199 has had a synergistic effect on positive NSCLC for EGFR mutations resistant to anti-EGFR inhibitors (tyrosine kinase inhibitor) [22, 23], based on these studies, our research team proposed that ABT-199 could also exhibit a synergistic effect in combination with ALKi, our results evidence that there was synergistic effect in WT and G1202R mutant cell models, while C1156Y and L1196M exhibited good combination with crizotinib, ceritinib and alectinib.

## Conclusions

The expression of the BCL2 protein was heterogeneous, WT and G1202R cell models showed high expression while C1156Y and L1196M presented an expression close to baseline. These results were in line to the pharmacological susceptibility because the models that overexpressed BCL2 showed resistance to ABT-199 and those that had an expression close to baseline were sensitive.

The bioinformatics findings showed that ABT-199 could interact with mutated ALK proteins, however, this interaction is not enough to be able to compete with ceritinib in WT model, on the other hand, in the WT and G1202R models, ABT-199 shows a higher binding energy score than crizotinib and alectinib proposing it as its main competitor, however, our in vitro assays point to drug resistance to ABT-199. Finally, in the L1196M model, ABT-199 would also behave as a competitor to crizotinib and alectinib, suggesting it as a possible molecule that would interact with the ALK protein.

ALK^mutant^ with ABT-199 interaction contrasted by *in vitro* assays showed that ABT-199 decrease to the kinase activity in ALK (IC_50_ = 5.5 μM), in addition, ABT-199 also evidenced decreasing of ALK phosphorylation in WT and L1196M mutant, while in combination with ALKi also exhibited decreasing of ALK phosphorylation in WT and C1156Y, in L1196M not showed decreasing of phosphorylation with ceritinib, and ultimately, in G1202R not shown significative differences.

Finally, a wide range of synergy was evidenced in the WT and G1202R models when ABT-199 was combined with ALKi, while the C1156Y and L1196M models showed good combinatorial activity in combination with crizotinib and alectinib and additivity with ceritinib.

In summary we conclude that the inhibition of BCL2 protein could be purposed as a second therapeutic target in addition to ALK for EML4-ALK cell models in NSCLC.

## Supporting information

S1 Fig BCL2 protein keeps their expression in different conditions treatment nevertheless each EML4-ALK cell modells exhibit variable expression. A) Western blots analysis of BCL2 expression in four EML4-ALK cell models. B) Bar graph exhibit variable BCL2 expression in all four EML4-ALK cell models but keep their expression in different condition treaments (ns: with no significative differences).
(TIF)

S2 Fig ALK inhibitors (Positive control) and ABT199 (Proposed binding) in complex with the ALK active side. In this figure we can see that ABT199 as ALK inhibitors can interact with the active side of ALK protein, depite of ABT199 is a large molecule, it can fit into the pockect as ALK inhibitors crizotinib, ceritinib and alectinib.
(TIF)

S3 Fig Negative (Prednisone) and basal (ATP) control compounds binding to ALK protein. In this figure we can see a Blind molecular docking of Prednisone with ALK protein. The outcomes show that prednisne can interact with ALK out of active site, the binding energy exhibited a stronger negative value (-451.12 Kcal/mol), being considered as negative control in the proces of molecular docking. On the other hand, ATP molecule, a natural ligand in the phosphorylation pathway we can see that binds to ALK active side with a binding energy of 30.67 Kcal/mol.
(TIF)

S4 Fig ALK^wt^–ABT199 interactions. Interactions between ALK^WT^ (shown in blue) and ABT199 (shown in orange). Visualized through a sticks format, the amino acids surrounding the ligand. Additionally, distinct colors of the amino acid highlight positions typically susceptible to mutation within the ALK protein structure. In addition, we showed hydrophobic and hydrogen bond interactions.
(TIF)

S5 Fig ALKC1156Y –ABT199 interactions. Interactions between ALKC1156Y (shown in blue) and ABT199 (shown in orange). Visualized through a sticks format, the aminoacids surrounding the ABT199 ligand. Additionally, in yellow the C1156Y mutated aminoacid. In addition, we showed hydrophobic and hydrogen bond interactions.
(TIF)

S6 Fig ALKL1196M –ABT199 interactions. Interactions between ALKL1196M (shown in blue) and ABT199 (shown in orange). Visualized through a sticks format, the aminoacids surrounding the ABT199 ligand. Additionally, in green the L1196M mutated aminoacid. In addition, we showed hydrophobic and hydrogen bond interactions.
(TIF)

S7 Fig ALKG1202R –ABT199 interactions. Interactions between ALKG1202R (shown in blue) and ABT199 (shown in orange). Visualized through a sticks format, the aminoacids surrounding the ABT199 ligand. Additionally, in red the G1202R mutated aminoacid. In addition, we showed hydrophobic, hydrogen bond, and π-Cation interactions.
(TIF)

S8 Fig Western blot analysis of drug combinations. Ba/F3 cells expressing wild-type (A) or L1196M (B), C1156Y (C) and G1202R (D) mutant EML4/ALK were treated for 48 hours with crizotinib, alectinib and venetoclax as single agents or in combinations, and ALK phosphorylation was determined, along with total ALK expression. Actin is shown as a loading control. (E) Effects of ceritinib/venetoclax combination on ALK+ Ba/F3 cells.
(TIF)

S1 Table The binding energy values of ATP, ALK inhibitors and ABT-199 to ALK were obtained from AlphaFold and modeled protein structures. Supporting Information contains the following supplementary tables: A) We can see ligand (ATP, crizotinib, ceritinib, alectinib, and ABT-199) binding energies calculated to ALK from alpha fold, B) from modelled protein, and C) final consensus where we exhibit media and SD values; and ANOVA test analysis.
(TIF)

S1 Data (RAR)
