# Supplementary figures and images for: Inhibition of the anti-apoptotic protein BCL2 in EML4-ALK cell models as a second proposed therapeutic target for non-small cell lung cancer
Source: PLoS One. 2025 Jan 21;20(1):e0308747. doi: 10.1371/journal.pone.0308747 (PMC11750102; doi:10.1371/journal.pone.0308747)

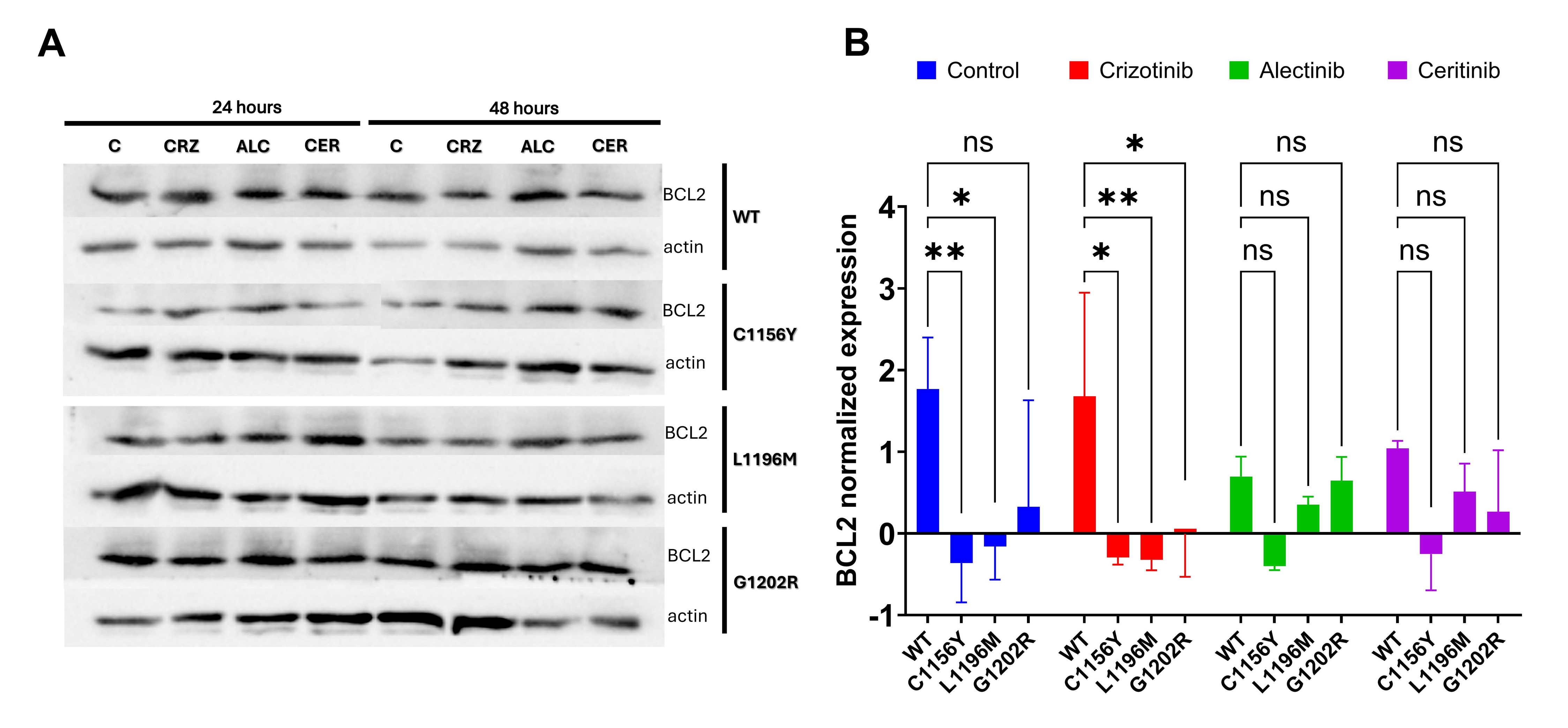

Supplement: S1 Fig — A) Western blots analysis of BCL2 expression in four EML4-ALK cell models. B) Bar graph exhibit variable BCL2 expression in all four EML4-ALK cell models but keep their expression in different condition treaments (ns: with no significative differences). (TIF) [file pone.0308747.s001.tif]

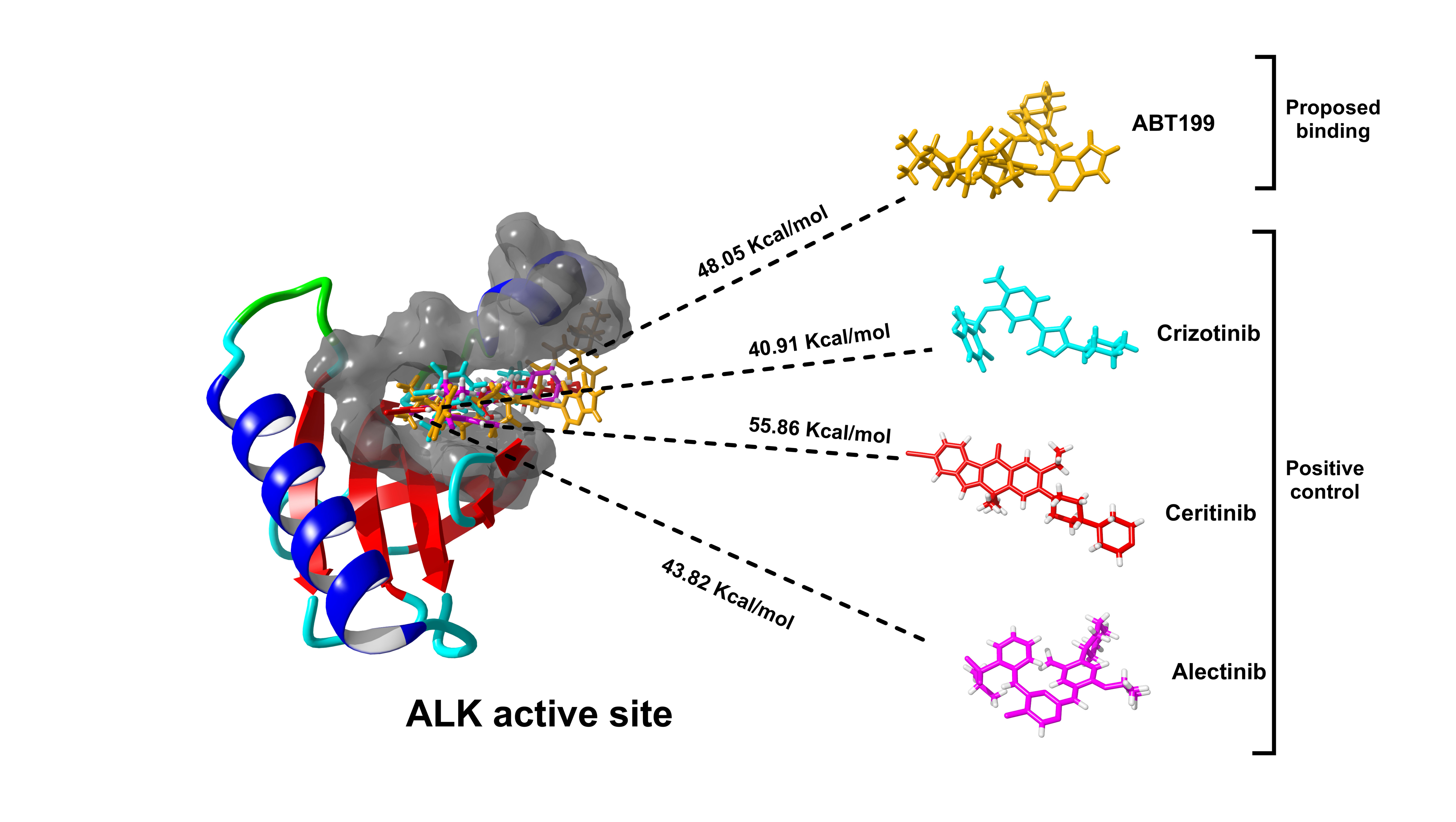

Supplement: S2 Fig — In this figure we can see that ABT199 as ALK inhibitors can interact with the active side of ALK protein, depite of ABT199 is a large molecule, it can fit into the pockect as ALK inhibitors crizotinib, ceritinib and alectinib. (TIF) [file pone.0308747.s002.tif]

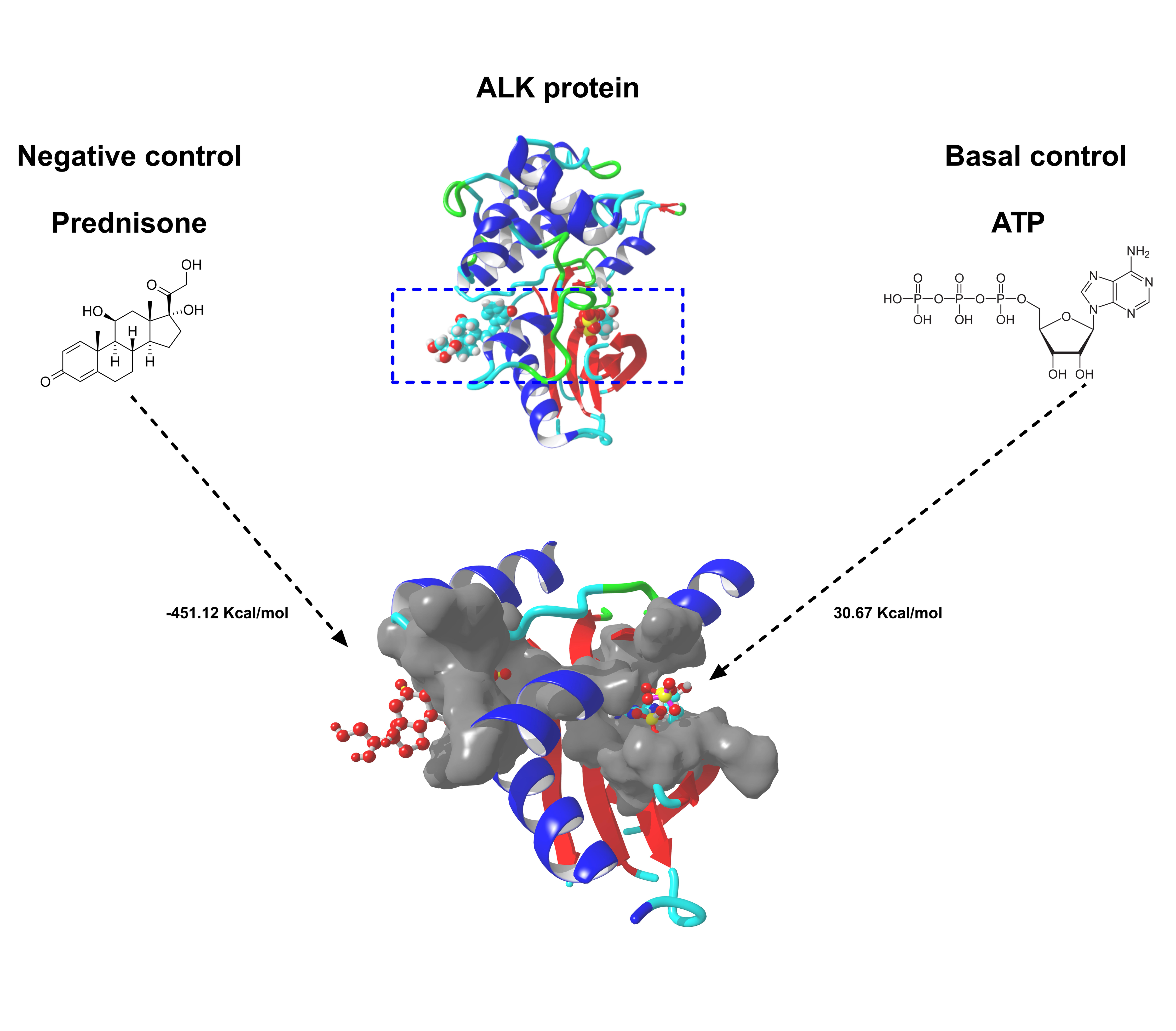

Supplement: S3 Fig — In this figure we can see a Blind molecular docking of Prednisone with ALK protein. The outcomes show that prednisne can interact with ALK out of active site, the binding energy exhibited a stronger negative value (-451.12 Kcal/mol), being considered as negative control in the proces of molecular docking. On the other hand, ATP molecule, a natural ligand in the phosphorylation pathway we can see that binds to ALK active side with a binding energy of 30.67 Kcal/mol. (TIF) [file pone.0308747.s003.tif]

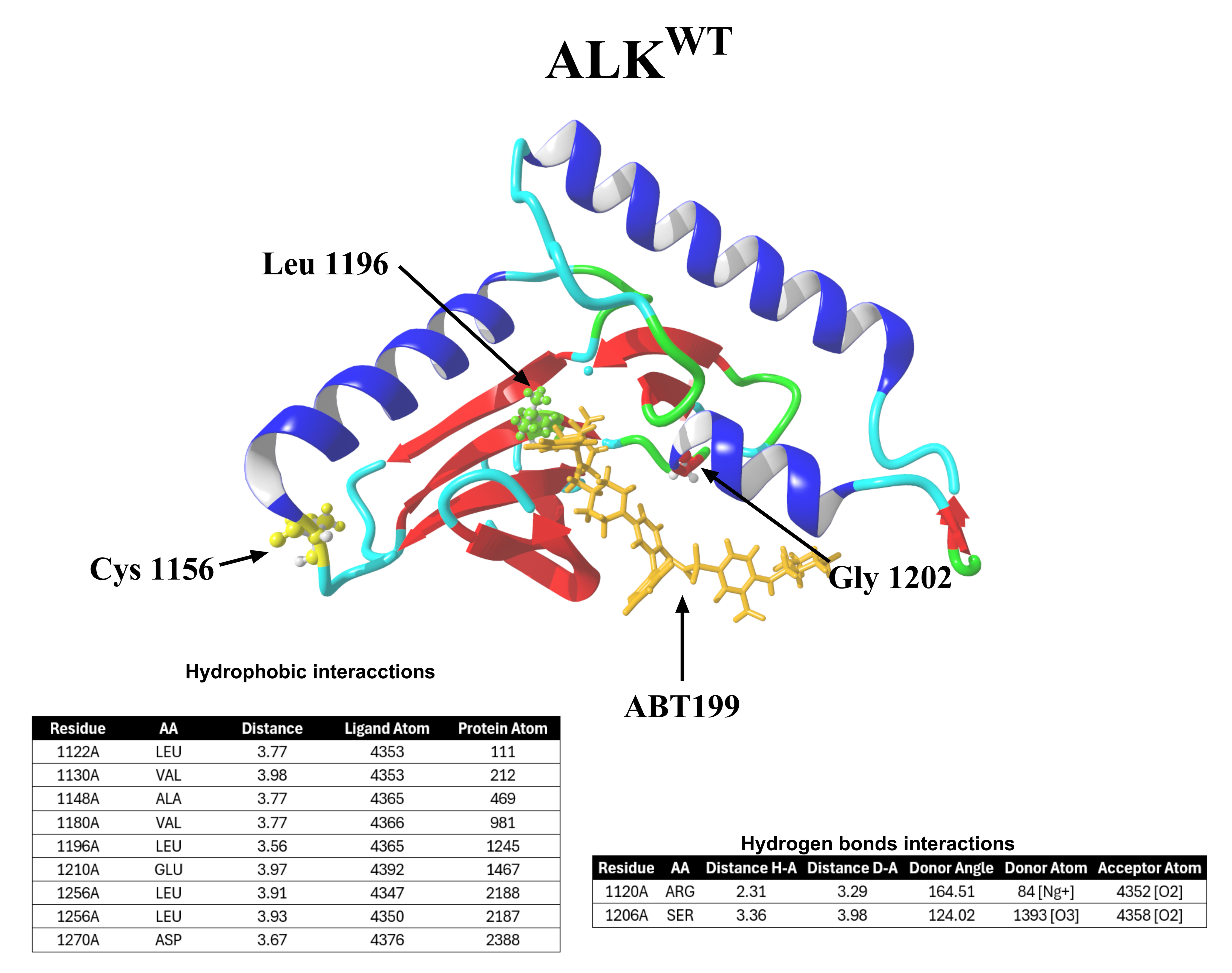

Supplement: S4 Fig — Interactions between ALKWT (shown in blue) and ABT199 (shown in orange). Visualized through a sticks format, the amino acids surrounding the ligand. Additionally, distinct colors of the amino acid highlight positions typically susceptible to mutation within the ALK protein structure. In addition, we showed hydrophobic and hydrogen bond interactions. (TIF) [file pone.0308747.s004.tif]

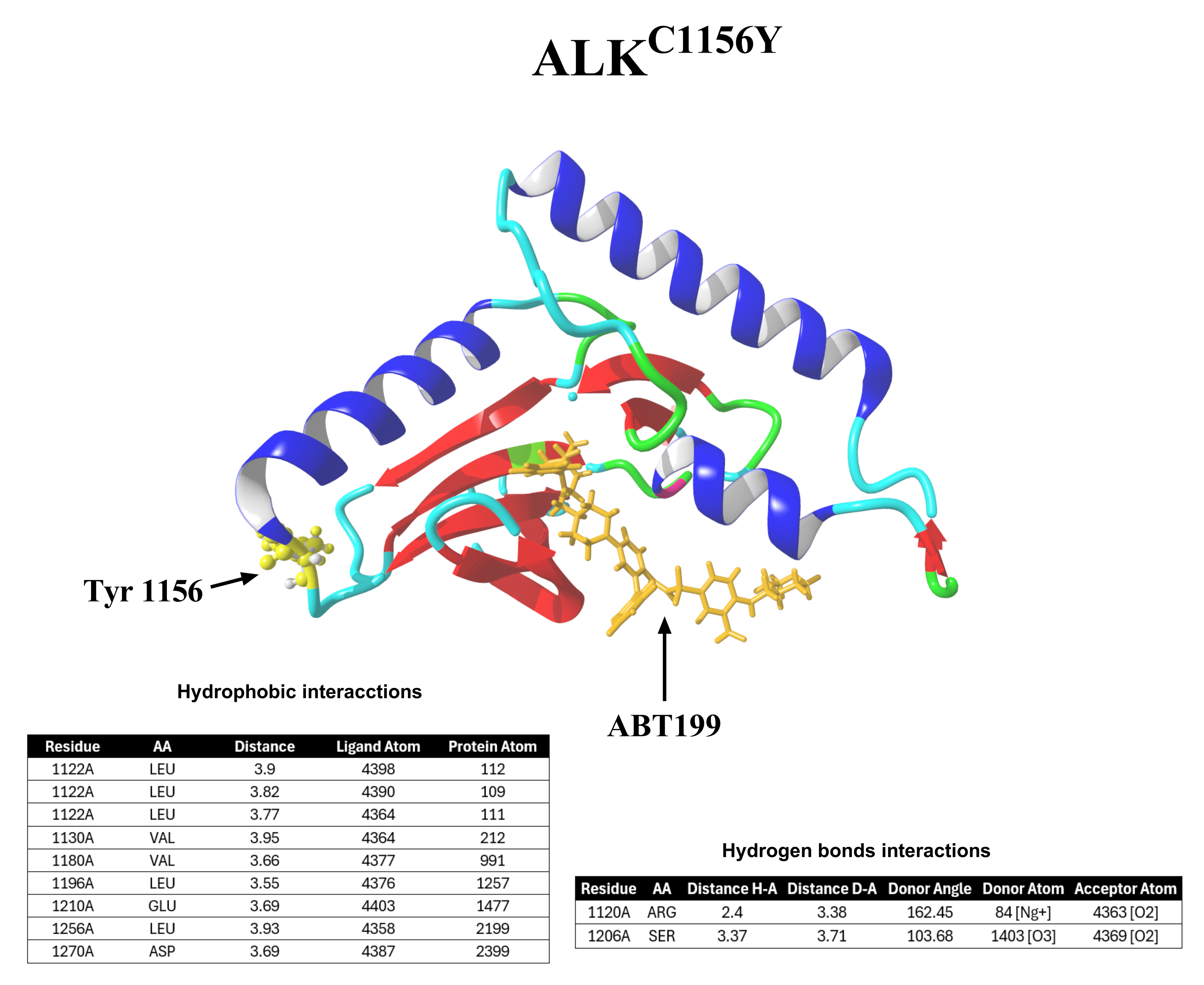

Supplement: S5 Fig — Interactions between ALKC1156Y (shown in blue) and ABT199 (shown in orange). Visualized through a sticks format, the aminoacids surrounding the ABT199 ligand. Additionally, in yellow the C1156Y mutated aminoacid. In addition, we showed hydrophobic and hydrogen bond interactions. (TIF) [file pone.0308747.s005.tif]

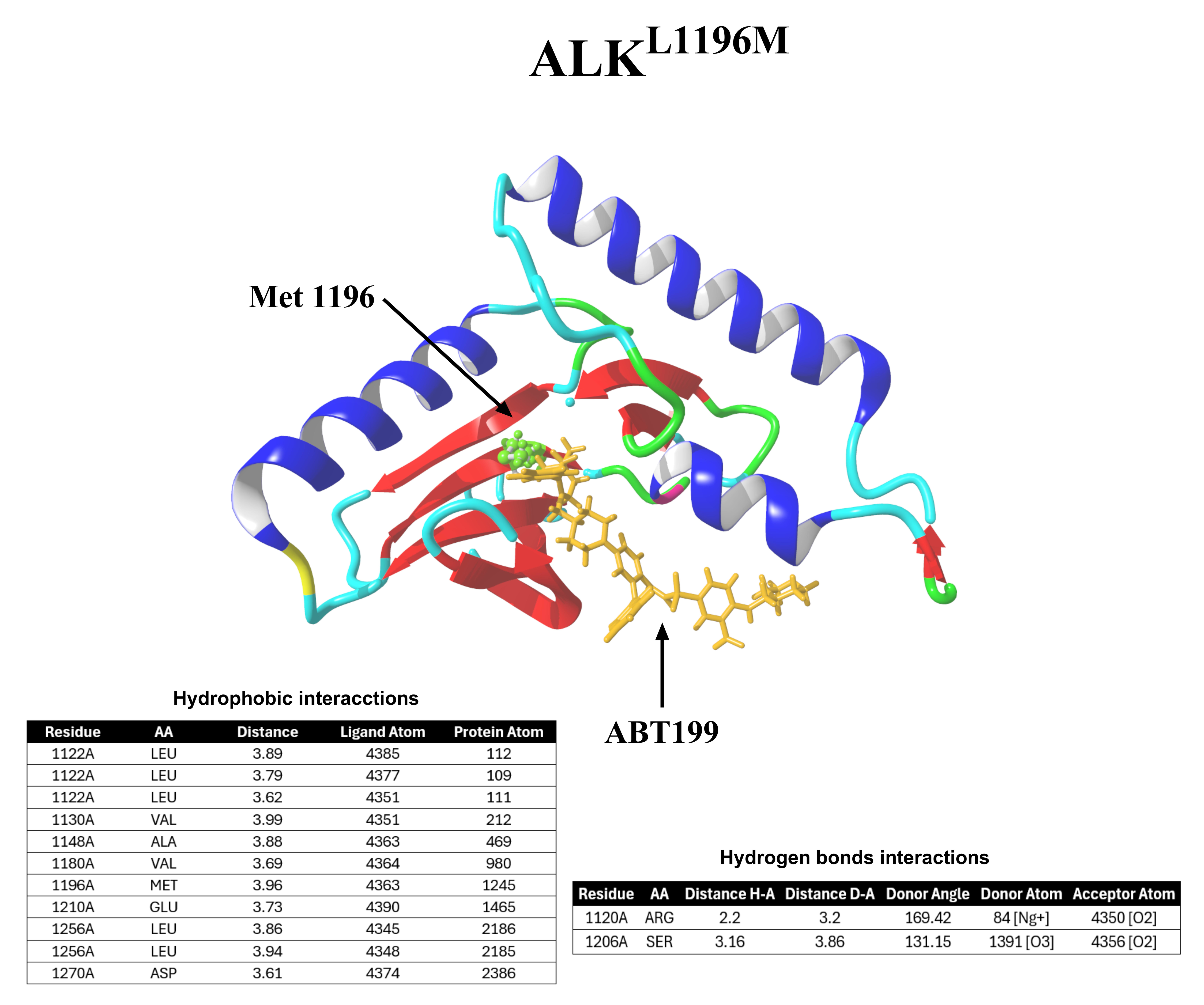

Supplement: S6 Fig — Interactions between ALKL1196M (shown in blue) and ABT199 (shown in orange). Visualized through a sticks format, the aminoacids surrounding the ABT199 ligand. Additionally, in green the L1196M mutated aminoacid. In addition, we showed hydrophobic and hydrogen bond interactions. (TIF) [file pone.0308747.s006.tif]

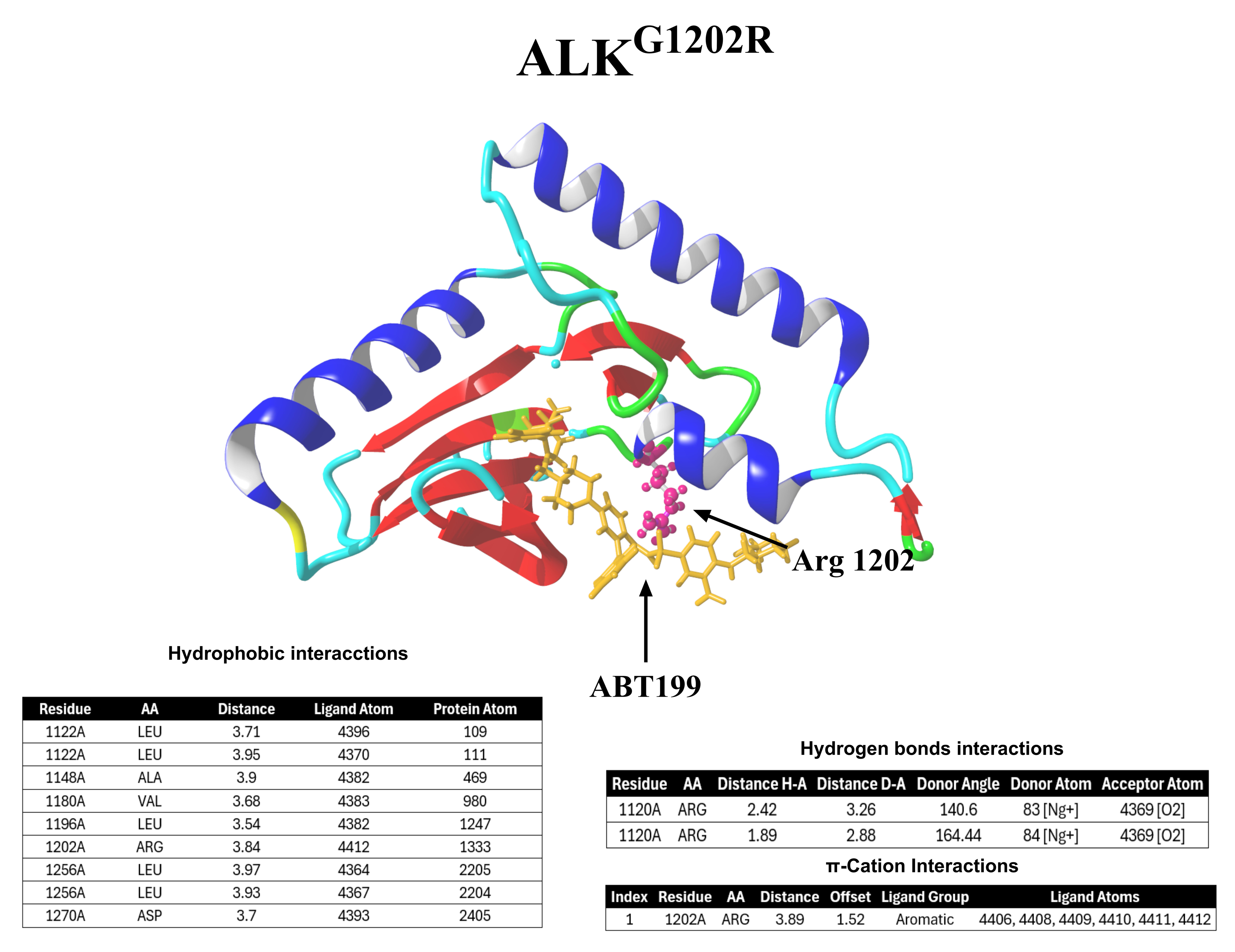

Supplement: S7 Fig — Interactions between ALKG1202R (shown in blue) and ABT199 (shown in orange). Visualized through a sticks format, the aminoacids surrounding the ABT199 ligand. Additionally, in red the G1202R mutated aminoacid. In addition, we showed hydrophobic, hydrogen bond, and π-Cation interactions. (TIF) [file pone.0308747.s007.tif]

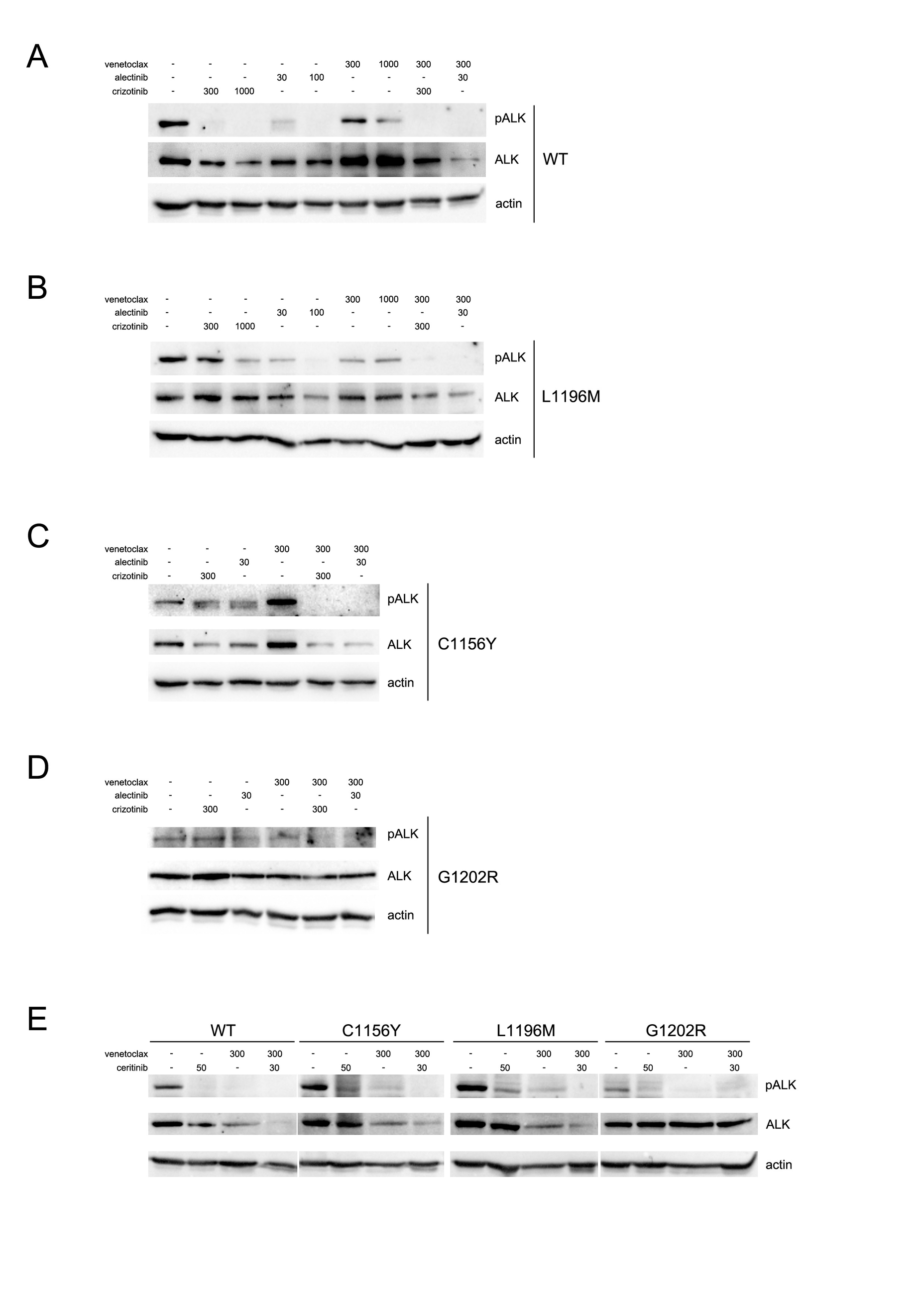

Supplement: S8 Fig — Ba/F3 cells expressing wild-type (A) or L1196M (B), C1156Y (C) and G1202R (D) mutant EML4/ALK were treated for 48 hours with crizotinib, alectinib and venetoclax as single agents or in combinations, and ALK phosphorylation was determined, along with total ALK expression. Actin is shown as a loading control. (E) Effects of ceritinib/venetoclax combination on ALK+ Ba/F3 cells. (TIF) [file pone.0308747.s008.tif]

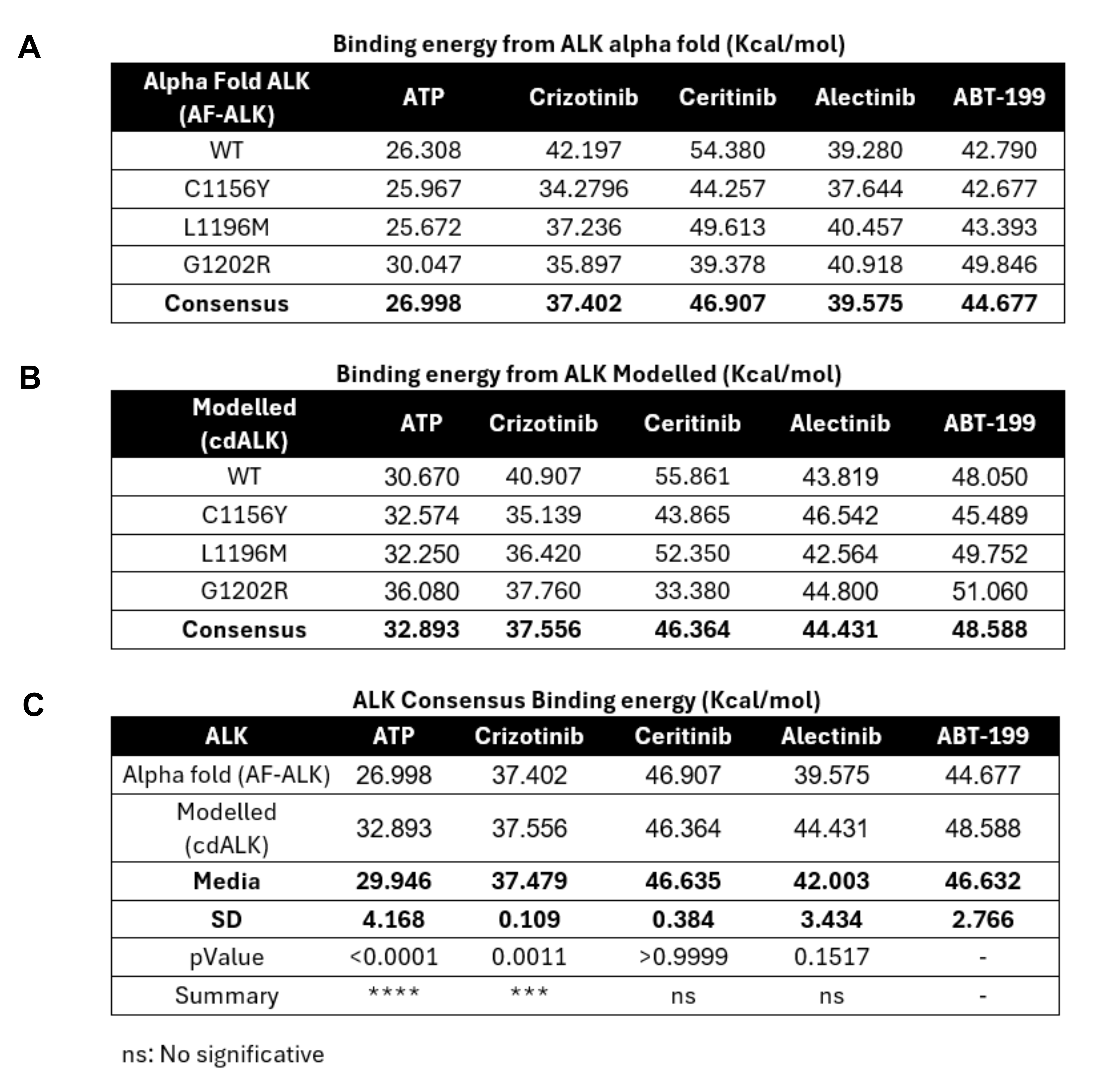

Supplement: S1 Table — Supporting Information contains the following supplementary tables: A) We can see ligand (ATP, crizotinib, ceritinib, alectinib, and ABT-199) binding energies calculated to ALK from alpha fold, B) from modelled protein, and C) final consensus where we exhibit media and SD values; and ANOVA test analysis. (TIF) [file pone.0308747.s009.tif]
